# Nail‐Gun Injury of the Thumb: Think Before Pulling

**DOI:** 10.1002/jgf2.70152

**Published:** 2026-07-11

**Authors:** Junya Shimamoto, Masahiro Kato, Hirotaka Yasui

**Affiliations:** ^1^ Department of General Medicine Ehime Prefectural Minamiuwa Hospital Ehime Japan; ^2^ Department of Orthopedics Ehime Prefectural Minamiuwa Hospital Ehime Japan

A man in his 30s presented to our hospital after accidentally shooting a nail through his right thumb while operating an automatic nail gun at a construction site (Figure 1A). The nail penetrated the thumb through a protective work glove. On examination, distal sensation, circulation, and active motion were preserved. Although the injury appeared severe, plain radiographs obtained in two orthogonal views demonstrated that the nail traversed the soft tissue without involvement of the distal phalanx or the distal interphalangeal joint (Figure 2A,B).

**FIGURE 1:**
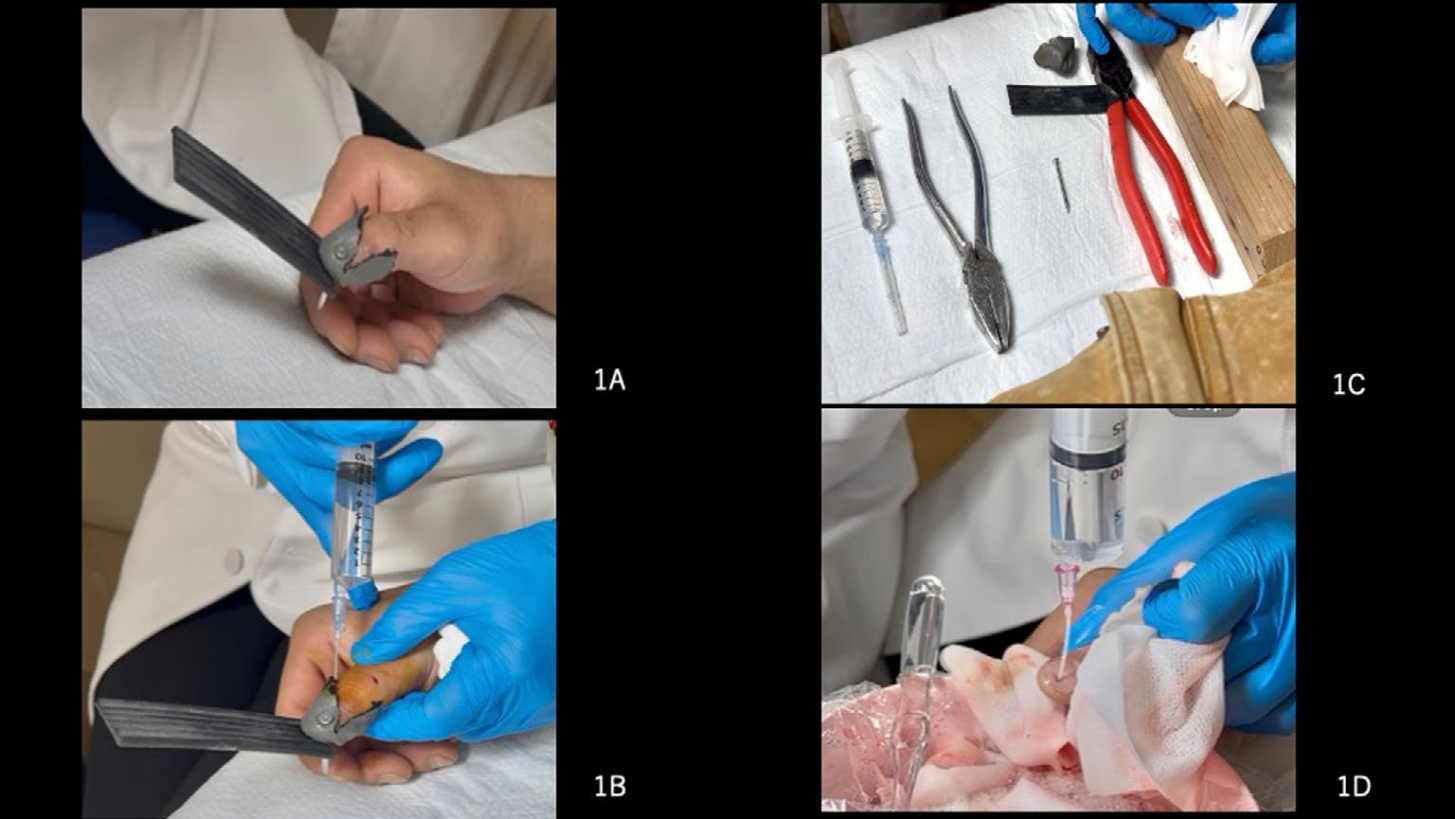
Management of a nail‐gun injury to the right thumb. (A) Nail penetrating the right thumb through a protective work glove (B) Digital nerve block using 1% lidocaine before extraction (C) Extracted nail and instruments used for bedside removal (D) Irrigation of the penetrating wound after nail removal.

**FIGURE 2:**
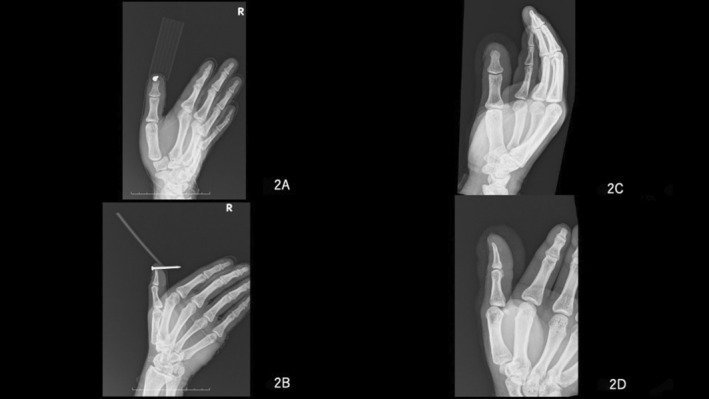
Orthogonal radiographic assessment of a nail‐gun injury to the right thumb (A) Anteroposterior radiograph demonstrating penetration of the thumb by a nail without obvious involvement of the distal phalanx. (B) Lateral radiograph showing the trajectory of the nail through the soft tissue and confirming the absence of distal interphalangeal joint penetration. (C) Postextraction lateral radiograph demonstrating complete removal of the nail without retained radiopaque foreign bodies. (D) Postremoval anteroposterior radiograph demonstrating no fracture or osseous injury of the distal phalanx.

After radiographic assessment, the glove around the nail was carefully cut away while preserving the portion entrapped by the nail. A digital nerve block was then performed using 1% lidocaine (Figure 1B). As the radiograph indicated a smooth nail without barbs, it was successfully extracted retrogradely under stabilization of the thumb (Figure 1C), and the wound was thoroughly irrigated with copious amounts of water (Figure 1D). Follow‐up radiographs confirmed complete removal of the nail without retained radiopaque foreign bodies (Figure 2C,D). Tetanus toxoid was administered because the injury represented a contaminated puncture wound. Oral amoxicillin‐clavulanate was prescribed for 7 days to provide anti‐staphylococcal coverage and reduce the risk of wound infection. The patient was followed for one month and recovered without infection, neurovascular deficit, or functional impairment.

Nail‐gun injuries commonly involve the hand and may be associated with fractures, tendon injuries, neurovascular injuries, and intra‐articular penetration. Although nail‐gun nails are available in several designs, the presence of nail barbs is particularly important because it may alter the extraction strategy and the need for orthopedic or hand surgical consultation [1]. At least two orthogonal radiographic views should be obtained to assess bony involvement, joint penetration, retained foreign material, and nail barbs that may influence the extraction strategy [2]. In the present case, a single anteroposterior view could have overestimated the depth of penetration. Orthogonal views demonstrated that the nail passed through soft tissue only, allowing safe bedside removal without referral for operative exploration. Previous case series have demonstrated that many nail‐gun injuries can be managed successfully with local wound care and extraction after careful radiographic assessment, provided that neurovascular, tendinous, and articular structures are not involved [3]. The presence of barbs may alter the direction of extraction because retrograde removal can cause additional soft‐tissue injury [4]. According to the published management algorithm [1], our patient had no evidence of neurovascular injury, tendon injury, unstable fracture, or intra‐articular penetration and was therefore appropriately managed with local extraction, irrigation, tetanus prophylaxis, antibiotics, and close follow‐up.

In primary care, emergency, and rural settings, clinicians are often required to perform minor surgical procedures, including digital nerve block, foreign‐body removal, wound irrigation, and tetanus prophylaxis. This case highlights the importance of combining procedural skills with appropriate imaging assessment. Clinicians should also remember that radiolucent foreign bodies, such as wood, bamboo [5], clothing fibers, and glove fragments, may not be visible on plain radiographs. The retained glove material surrounding the nail also represented a potential source of contamination and foreign‐body retention. Careful removal of entrapped clothing or glove fragments before extraction may reduce the risk of retained debris and subsequent wound infection. Orthopedic or hand surgical consultation should be considered when neurovascular injury, tendon injury, unstable fracture, intra‐articular involvement, significant contamination, or deeply embedded or barbed nails are suspected.

## Author Contributions

**Hirotaka Yasui:** writing – review and editing, supervision. **Masahiro Kato:** conceptualization, investigation, writing – review and editing. **Junya Shimamoto:** conceptualization, writing – original draft, investigation, validation, methodology, resources, visualization.

## Ethics Statement

Ethics approval was waived by the institutional review board because of the design of this study.

## Consent

Written informed consent was obtained from the patient for publication of this case report and accompanying images.

## Conflicts of Interest

The authors declare no conflicts of interest.

## Data Availability

The data that support the findings of this study are available upon request from the corresponding author. The data are not publicly available due to privacy or ethical restrictions.
